# Crosslinking of membrane CD13 in human neutrophils mediates phagocytosis and production of reactive oxygen species, neutrophil extracellular traps and proinflammatory cytokines

**DOI:** 10.3389/fimmu.2022.994496

**Published:** 2022-11-10

**Authors:** Erandi Pérez-Figueroa, Pablo Álvarez-Carrasco, Enrique Ortega

**Affiliations:** Department of Immunology, Instituto de Investigaciones Biomédicas, Universidad Nacional Autónoma de México, Ciudad Universitaria, Ciudad de Mexico, Mexico

**Keywords:** Aminopeptidase N, phagocytic receptor, ROS - reactive oxygen species, NETs, neutrophil membrane receptor

## Abstract

Aminopeptidase N, or CD13, is a cell membrane ectopeptidase highly expressed in myeloid cells. Through its enzymatic activity, CD13 regulates the activity of several bioactive peptides, such as endorphins and enkephalins, chemotactic peptides like MCP-1 and IL-8, angiotensin III, bradikinin, etc. In recent years, it has been appreciated that independently of its peptidase activity, CD13 can activate signal transduction pathways and mediate effector functions such as phagocytosis and cytokine secretion in monocytes and macrophages. Although neutrophils are known to express CD13 on its membrane, it is currently unknown if CD13 can mediate effector functions in these cells. Here, we show that in human neutrophils CD13 can mediate phagocytosis, which is dependent on a signaling pathway that involves Syk, and PI3-K. Phagocytosis mediated by CD13 is associated with production of reactive oxygen species (ROS). The level of phagocytosis and ROS production mediated by CD13 are similar to those through FcγRIII (CD16b), a widely studied receptor of human neutrophils. Also, CD13 ligation induces the release of neutrophil extracellular traps (NETs) as well as cytokine secretion from neutrophils. These results support the hypothesis that CD13 is a membrane receptor able to activate effector functions in human neutrophils.

## Introduction

Neutrophils are the most abundant leukocytes in human blood, and are the first cells to be recruited to an inflammatory site. Upon arrival at the inflamed tissue, they act as powerful effector cells involved in elimination of invading pathogens by multiple mechanisms, including phagocytosis, generation of reactive oxygen species (ROS), release of neutrophil extracellular traps (NETs), degranulation, and release of proinflammatory mediators (1, 2). Furthermore, in addition to their important effector role against pathogenic microbes, in recent years it has become increasingly evident that these cells participate in shaping adaptive immune responses as well as in several pathological conditions (reviewed in (3–5). Neutrophil responses are controlled by a wide variety of membrane receptors, including pattern recognition receptors such as TLRs and C-lectin receptors, G-protein coupled receptors, complement receptors, receptors for the Fc fraction of immunoglobulins (FcRs), etc. (6, 7).

Aminopeptidase N (E.C.3.4.11.2), also known as CD13 or APN, is a transmembrane zinc-dependent ectopeptidase expressed in different cells and tissues such as kidney, intestine, liver and placenta, as well as fibroblasts and endothelial cells. Structurally, CD13 is a heavily glycosylated type II membrane protein with a large extracellular domain that contains the active site, a single transmembrane region, and a short cytoplasmic tail of 8 amino acids. Among hematopoietic cells, CD13 is highly expressed in myeloid cells: monocytes, macrophages, dendritic cells and neutrophils, and has been used as a myeloid marker (8–10). By virtue of its peptidase activity, CD13 cleaves N-terminal neutral residues of small peptides, and through this activity it modulates the action of several bioactive peptides, such as endorphins and enkephalins, chemotactic peptides as MCP-1 and IL-8, angiotensin III, bradikinin, etc. However, several additional functions have been attributed to CD13, leading to it being considered a moonlighting protein (11). Thus, CD13 is a receptor for group 1 coronavirus, including the human α-coronavirus HCoV-229E (12, 13), and participates in infection of human cells by cytomegalovirus (14). It has also been shown that CD13 acts as an adhesion molecule that induces homotypic aggregation (15–17), is involved in migration and invasion of cancer cells, participates in angiogenesis (18), and regulates recycling of β1-integrins (19). Importantly, it has been demonstrated that CD13 is able to induce signal transduction pathways inside the cell (17, 20, 21). The soluble form of CD13 has been reported to play an important role in angiogenesis and inflammation (22, 23), and CD13 has been recently proposed as a potential therapeutic target in inflammatory diseases (24).

While some of these functions depend on the aminopeptidase activity of CD13, others do not require its catalytic activity, and it has been proposed that these depend on structural features independent of its active site (25). Inhibition of CD13 enzymatic activity by pharmacological inhibitors, by mutation of residues in the active site that are essential for enzymatic activity, or by anti-CD13 antibodies that inhibit the enzymatic activity, has given evidence that at least three types of functions of CD13 do not require its enzymatic activity: *i*) as a viral receptor (14, 26, 27) *ii*) as an adhesion molecule (15, 16), and *iii*) as a signaling molecule (17, 21, 28, 29). Interestingly, all of these functions rely on CD13 crosslinking on the cell membrane by either antibodies or viral ligands, which presumably bring about signal transduction. In monocytic cells, crosslinking of CD13 by anti-CD13 antibodies induces Ca2+ mobilization associated with activation of ERK1/2, JNK and p38 kinases (20, 29). Also, in U937 monocytic cells, crosslinking of CD13 by monoclonal antibodies induces phosphorylation of proteins such as focal adhesion kinase (FAK), Src family protein kinases and the MAPK ERK 1/2, as well as cytoskeleton rearrangements, and all these are associated with phosphorylation of Tyr6 in the cytoplasmic portion of the molecule (17).

We have previously reported that CD13 is able to mediate phagocytosis in human monocyte-derived macrophages and THP-1 monocytic cells. Phagocytosis was found to be independent of CD13 enzymatic activity, but dependent on actin rearrangement, PI3-K activation, and partially dependent on Syk activation. Moreover, it was shown that after transfection of nonphagocytic HEK293 cells with human CD13 (hCD13), these cells acquire the ability to phagocytize particles bound through CD13 (30).

Human neutrophils are known to express CD13 on the membrane (10), but the involvement of CD13 in neutrophil functions has been evaluated only rarely. It has been reported that CD13 regulates apoptosis of human neutrophils induced by TNF-α (31). CD13 is also involved in IL-8-induced neutrophil migration in collagen gels, as some anti-CD13 antibodies inhibited this phenomenon. Anti-CD13 antibodies also induced significant homotypic aggregation of neutrophils, which was dependent on CD13 crosslinking and activation of PI3-K and ERK 1/2 (32). However, the role of CD13 in effector functions of neutrophils such as phagocytosis, ROS production and NET formation, has not been studied. In this paper, we show that in human neutrophils, crosslinking of membrane CD13 mediates phagocytosis and production of reactive oxygen species, neutrophil extracellular traps and proinflammatory cytokines. These findings further support the contention that CD13 can be considered as an innate immune receptor able to mediate effector functions in distinct types of human myeloid cells.

## Materials and methods

### Reagents and antibodies

Fetal bovine serum (FBS), RPMI-1640 medium, sodium pyruvate solution, MEM non-essential amino acids solution, L-glutamine, penicillin, and streptomycin were purchased from Gibco Life Technologies (Carlsbad, CA, USA). Lymphoprep was from Axis-Shield PoC AS (Oslo, Norway). Carboxy-H2DFFDA and carboxyfluorescein succinimidyl ester (CFSE) were from Molecular Probes Life Technologies (Eugene, OR, USA). Sulfo-NHS-Biotin was from Thermo Scientific (Waltham, MA, USA); Streptavidin was from Calbiochem (San Diego, CA, USA), and bovine serum albumin (BSA) was from Sigma (St. Louis, MO, USA). Recombinant Human Interleukin 8 (rhIL-8) was from PeproTech (Rocky Hill, NJ, USA) and Phorbol-12-Myristate-13-Acetate (PMA, synthetic, ≥98.0%) was from Sigma (St. Louis, MO, USA). Culture media (RPMI-1640, Gibco) was supplemented with 2% heat-inactivated FBS and 1 mM sodium pyruvate, 0.1 mM non-essential amino acids solution, 0.1 mM L-glutamine, 100 U/mL penicillin, and 100 μg/mL streptomycin (complete media). Cultures were maintained in a humidified atmosphere at 37°C with 5% CO_2_. Murine monoclonal anti-hCD13 (clone 452, IgG1) was purified in our laboratory from culture supernatants of the hybridoma, kindly donated by Dr. Meenhard Herlyn (Wistar Institute of Anatomy and Biology, Philadelphia, PA, USA). Murine monoclonal anti- hCD13 (mAb C, IgG1) was produced and purified in our laboratory as described (33). Murine monoclonal IgG2a anti-human FcγRII (clone IV.3) mAb was purified in our laboratory from supernatants of the corresponding hybridoma obtained from American Type Culture Collection. Fab fragments of the antibodies were prepared with immobilized Ficin (Pierce, Rockford, IL, USA) following the manufacturer’s instructions. Anti-CD16b antibody labeled with Phycoerithrin (PE) (anti-human CD16b, Workshop number IV N404, BD-Pharmingen) was a kind gift of Dr. C. Maldonado-Bernal (Hospital Infantil de México, Mexico City, Mexico). PE-Mouse anti-phospho-Syk antibody pY348 (Cat. 558529) was from BD Biosciences.

Biotin-F(ab′)2 fragments of goat anti-mouse IgG (H + L) were from Invitrogen and from Life Technologies (Eugene, OR, USA). Goat anti-mouse Ig-FITC, used as a secondary antibody for immunostaining, was from Invitrogen. Monoclonal mouse anti-human CD13 (clone WM-15) labeled with APC was from Biolegend. Monoclonal mouse anti-human CD16 (clone 3G8) was a kind gift from Dr Carlos Rosales, Instituto de Investigaciones Biomédicas, UNAM.

### Purification of human neutrophils

Human neutrophils were obtained from peripheral blood of healthy human volunteers. The experiments conducted with these cells followed a protocol approved by the Bioethics Committee of the Instituto de Investigaciones Biomédicas – UNAM. All volunteers gave written informed consent in accordance with the Declaration of Helsinki. Neutrophils were purified by density gradient centrifugation in two steps (34, 35). Briefly, blood (10 mL) was collected in BD Vacutainer^®^ tubes with Lithium/Sodium Heparin (158USP). The collected blood was placed in a 15-mL tube with 2 mL of 6% Dextran solution in PBS. Erythrocytes were allowed to settle for 45 min at room temperature. After this period of time, the upper layer (containing plasma and leukocytes) was taken and carefully placed on top of 5 mL of Lymphoprep ™ (Diatrizoate sodium, 1.077 g/mL) at 4°C in a 15-mL tube. The tube was centrifuged for 20 min at 515 x*g* at 4°C. The upper layers (plasma, mononuclear cells and Lymphoprep) were removed, and the cell pellet was resuspended in 10 mL of a hypotonic solution (0.2% NaCl) supplemented with 1% BSA for exactly 1 min. The 10 mL of cell suspension were transferred to a 50 mL tube which contained 10 mL of hypertonic solution (1.2% NaCl/1% BSA) and the suspension was gently mixed. Finally, the tubes were centrifuged for 5 min at 300 x*g*, the supernatant was decanted, and the cell pellet was resuspended in Hanks’ solution (HBSS) (NaCl 0.137 M; KCl 0.054 M, glucose 0.1 g/100 mL, Na_2_HPO_4_ 0.25 mM, NaHCO_3_ 4.2 mM, KH_2_PO_4_ 0.44 mM, CaCl_2_ 1.3 mM, MgSO_4_ 1.0 mM, pH 7.4) at 4°C at a final concentration of 1x10^6^ cells/mL. Resulting cell suspensions contained > 95% neutrophils as evaluated by expression of CD16b.

### Determination of neutrophil CD13 expression

Expression of CD13 and CD16b on human neutrophils was evaluated with monoclonal antibodies specific for CD13 (WM15-APC from Bio Legend) and for CD16b (3G8-PE from BD Pharmigen). Cells (2x10^5^) suspended in FACS buffer (PBS with 2% FBS, 0.1% NaN_3,_ 5 mM EDTA) were incubated with the conjugated antibodies in flexible 96-well plates and protected from light for 30 min. After this time, the cells were washed with FACS buffer and fixed with paraformaldehyde (PFA) at 1% for 30 min. Finally, the samples were analyzed by flow cytometry (Attune^®^ Acoustic Focusing Cytometer Blue/Violet: 488 nm and 405 nm laser, Applied Biosystems Carlsbad, CA, USA). Data were analyzed using FlowJo Vx 7.0 software.

### Phagocytosis through CD13, FcγRII, or FcγRIIIb (Selective phagocytosis)

Sheep red blood cells (SRBCs) for phagocytosis assays were stained with CFSE, labeled with biotin, incubated with Streptavidin, and covered with biotinylated F(ab)´2 goat anti-mouse Ig as described previously by Mendoza–Coronel (36). Neutrophils (1x10^6^ cells/1 mL HBSS) were incubated with 10 µg of one of the following antibodies: Fab fragments of mAb452 (anti-CD13), Fab fragments of mAb C (anti-CD13), Fab fragments of mAb IV.3 (anti-FcγRII), mAb 3G8 (anti-CD16b), or without treatment (No Fab) for 30 minutes at 4°C, washed, and incubated with CFSE-labeled sheep erythrocytes covered with F(ab)´2 goat anti-mouse Ig (EBS-Fab) at a ratio of 1 neutrophil:20 EBS-Fab, at 37°C for 60 minutes. Non-internalized erythrocytes were lysed by hypotonic shock, and phagocytosis was quantified by flow cytometry (Attune Acoustic Focusing Cytometer; Applied Biosystems, Carlsbad, CA, USA). Data are expressed as the percentage of CFSE-positive cells.

To determine the effect of pharmacological inhibitors of kinases or actin polymerization on the phagocytosis, after incubation with the respective Fab fragments, neutrophils were incubated for 20 min at 37°C, with 5 µM cytochalasin D (an inhibitor of actin polymerization, CAS No. 22144-77-0), or 10 µM BAY (Syk Inhibitor IV, BAY 61-3606 - CAS No. 732938-37-8), or 20 µM LY294002 (a morpholine-containing compound that is a strong inhibitor of phosphoinositide 3-kinases, CAS No. 154447-36-6). (all from Sigma-Aldrich, St. Louis, MO, USA) or DMSO as vehicle control, and the phagocytosis assay was carried out as described above in the presence of the respective inhibitor.

### Inmunofluorescence

Selective phagocytosis assays were carried out as described above and after lysing the erythrocytes, an aliquot of the cells (approx. 1 x 10^4^ cells) was fixed with 1% paraformaldehyde, placed on a glass slide pre-treated with 0.01% poly-L-lysine (Sigma-Aldrich), and processed for immunofluorescense. Briefly, the fixed cells were allowed to air-dry on the glass slide. Once dry, the cells were permeabilized with 0.1% Triton X-100 in PBS for 30 min, and stained with 20 nM Phalloidine-rhodamine (Molecular Probes, Eugene,OR) for 45 min, and for 5 min with 20 nM DAPI (Invitrogene). Finally, the slide was washed and mounted with Fluoroshield^®^. Stained cells were observed with an epifluorescence Nikon Eclipse TE2000 microscope (Tokyo, Japan). Images were acquired with a Nikon DS-2MV camera and the Nikon NIS-Elements AR software.

#### Quantification of ROS production

Neutrophils (1x10^6^) were incubated with 10 µg of Fab fragments of anti CD13 (Fab 452 or Fab C), anti FcγRII (IV.3), mAb anti FcγRIIIb (CD16b), or no treatment (No Fab), for 30 min at 4°C in HBSS. After a brief centrifugation, the supernatant was discarded, and the cells were loaded with a cell-permeable ROS-sensitive fluorescent dye (5 mM carboxy-H2DFFDA) for 30 min at 37°C in HBSS. After washing, cells were transferred to wells (2 x 10^5^ cells/well) of black 96-well Immuno Plates (Thermo Scientific, Waltham, MA, USA). ROS production was induced by adding erythrocytes covered with F(ab’)_2_ fragments of goat anti-mouse Ig prepared as described above, but not loaded with CFSE, at a ratio of 1 cell:20 EBS-Fab, or with 20 nM PMA as a positive control. The cells mixed with the EBS-Fab were forced to the bottom of the well by a brief centrifugation to promote close contact between Neutrophils and erythrocytes. Each condition was analyzed in triplicate wells. Fluorescence intensity from oxidized carboxy-H2DFFDA was determined immediately (initial reading) and every 15 min thereafter, in a Cytation 3 Cell Imaging Multi-Mode Reader (BioTek, Winooski, VT, USA). Fluorescence readings were taken every 15 min during incubation at 37°C for 240 min.

### Induction of neutrophil extracellular traps

Kinetic analysis of NETs formation was conducted using the cell impermeable dsDNA dye SYTOX™ Green Nucleic Acid Stain (Invitrogen™). Human neutrophils (1 x 10^5^ cells/well) incubated or not with 10 µg of Fab fragments or complete anti-CD13 mAb 452, were seeded in 0.1 mL of RPMI medium supplemented with 2% FBS containing Sytox Green (500nM) in flat-bottom 96-well plates. NET formation was stimulated by addition of 10 µg of secondary F(ab’)_2_ fragments of goat anti-mouse Ig, with or without PMA (20 nM) or IL-8 (10 µg/mL). Every condition was assayed in triplicate wells. Blank consisted of medium RPMI-2% FBS-Sytox Green. The Sytox Green fluorescence was registered in a Cytation 3™ Cell Imaging Multi-Mode Reader from BioTek, at excitation and emission wavelengths of 485 and 528 nm respectively. Fluorescence readings were taken every 15 min during incubation at 37°C for 4 hours.

### Visualization of NETs by confocal microscopy

Glass cover slides treated with poly-L-lysine were placed in the bottom of 24-wells plates. Human Neutrophils (2 x 10^5^ cells/well) incubated of not with 10 µg of Fab fragments or complete anti-CD13 mAb 452, were seeded in 0.5 mL of RPMI medium supplemented with 2% FBS in individual wells. NET formation was stimulated by addition of 10 µg of secondary F(ab’)_2_ fragments of goat anti-mouse Ig, or with PMA (20 nM). Plates were incubated for 4 h at 37°C. After the incubation the RPMI medium was removed and the cells were fixed with 4% PFA in PBS for 10 minutes at room temperature. After this time, cells were washed 3 times with cold PBS and permeabilized with Triton X-100 0.2% for 5 minutes followed by washing 3 times with cold PBS. Finally, the cells were incubated with DAPI diluted 1: 1000 in PBS and incubated for 15 minutes in the dark. After washing 3 times with cold PBS, the coverslips were mounted with Fluoroshield (Sigma Chemical Co. St Louis, MO).

For experiments in which myeloperoxidase (MPO) and neutrophil elastase (NE) were visualized, before the incubation with DAPI, the cells were incubated with blocking buffer (Tween-20, 0.05% in PBS) for 30 minutes at 37°C, then incubated 1 hour at room temperature with the primary antibody for MPO (rabbit polyclonal anti-MPO, Abcam ab9535) or NE (rabbit polyclonal anti-NE, Abcam ab68672) diluted 1: 200 in PBS with 0.05% Tween-20 1% BSA. After this time, the cells were washed 3 times with cold PBS and incubated with the secondary antibody (Invitrogen, goat-anti-rabbit Ig-FITC No. 62-6511 for the NE and goat anti-rabbit Ig Alexa Fluor^®^ 594, ab150080 for the MPO) for 1 h at room temperature and washed 3 times with cold PBS. Finally, the cells were incubated with DAPI diluted 1: 1000 in PBS and incubated for 15 minutes in the dark, washed and mounted with Fluoroshield. Preparations were stored at 4°C until observation with a Nikon A1R+ STORM confocal microscope.

### Cytokine secretion

Neutrophils (1 x 10^6^) were incubated for 30 min at 4°C in RPMI medium with 2% FBS, with Fab fragments of anti-CD13 mAbs (FabC or Fab452), or anti-FcγRII (FabIV.3), or with mAb anti-CD16b (mAb 3G8), or left untreated. Cells were washed and subsequently treated with secondary F(ab’)_2_ fragments of goat anti-mouse Ig (10 ug) for 24 h at 37°C. Samples of cells not incubated with Fabs were treated with LPS (*E. coli* 100 ng/mL) as a control. Cell-free culture supernatants were collected and used to quantitatively measure IL-8, IL-1β, IL-6, IL-10 and TNF-α protein levels using a bead-based multiplex assay for flow cytometry (LEGENDplex™ Human Inflammation). All the necessary reagents were provided in the kit and prepared according to the manufacturer’s protocol. From every experiment, each condition was analyzed in duplicate, and the mean concentration of each cytokine from three independent experiments was calculated using a parameter logistic fitted curve generated from the standards using the program LEGENDplex V8.0.

### Statistical analysis

Statistical analysis was performed using GraphPad-Prism software (GraphPad, La Jolla, CA, USA). Data are expressed as the mean ± SEM. One-way ANOVA followed by Tukey’s *post hoc* test for comparisons between groups was used to compare among treatments. Statistical significance was considered with *p<* 0.05.

## Results

### Human neutrophils express CD13

**Figure 1:**
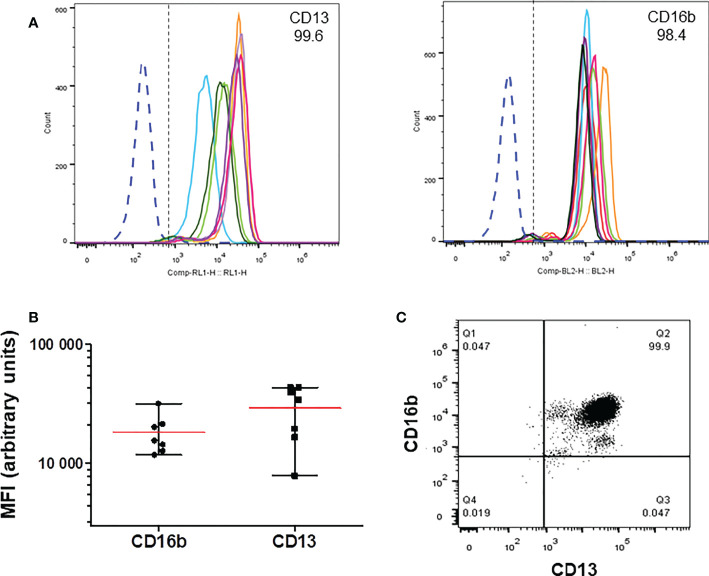
Expression of CD13 in human neutrophils. Human neutrophils were stained with anti-CD13-APC and anti-CD16b-PE, and analyzed by flow cytometry. **(A)** Representative histograms showing the expression of CD13 (left) and CD16b (right panel) in cells from individual donors. **(B)** Mean (± SEM) of the Mean Fluorescence Iintensity (MFI) values of the expression of CD13 and CD16b from the histograms shown in A **(C)** Representative dot plot of CD13 and CD16b expression in neutrophils from a single donor. More than 99.5 percent of cells express both markers.

Expression of CD13 on the membrane of neutrophils isolated from peripheral blood of human donors was evaluated by flow cytometry, along with the expression of CD16b as a marker of neutrophils. As shown in **Figure 1**, more than 99% of human neutrophils, identified by the expression of CD16b, were positive for expression of CD13. Both markers show variable levels among different individuals (**Figure 1A**), but the percentage of CD13 positive cells was never lower than 99%. The levels of CD13 expression were more variable than those of CD16b among different donors (**Figure 1B**), although in each and every donor virtually all neutrophils are CD16b+,CD13+ (**Figure 1C**).

### CD13 mediates phagocytosis in human neutrophils

**Figure 2:**
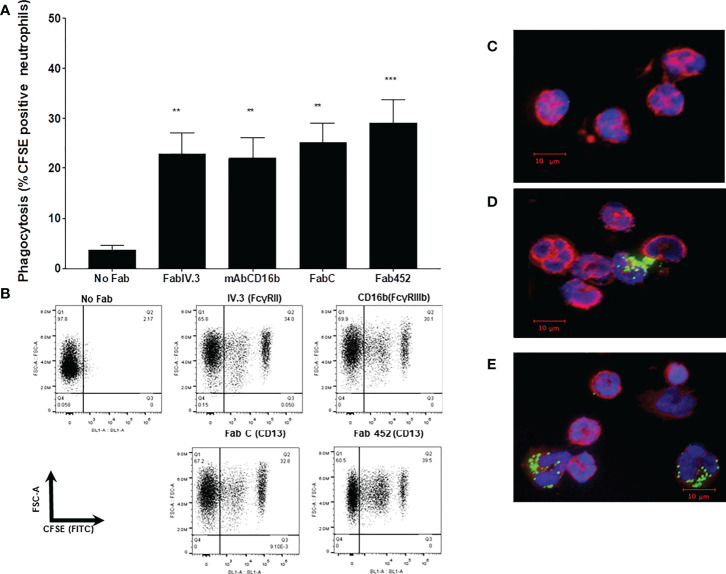
CD13 mediates phagocytosis in human neutrophils. Neutrophils were incubated with either Fab fragments of IV.3 (anti-FcγRII), mAb anti-CD16b (anti-FcγRIIIb), Fab C or Fab 452 (anti-CD13), or without antibody (No Fab), and phagocytosis of CFSE-labeled EBS-Fab was determined as described in materials and methods. **(A)** Average CFSE-positive cells from 11 independent experiments with cells from different donors. Bars represent the mean (± SEM) percentage of CFSE positive cells. **p < 0.01, ***p  < 0.001, relative to No Fab. **(B)** Representative dot plots of a single experiment after incubation at 37°C and lysis of non-internalized erythrocytes. FSC-A: Forward-scatter-area. **(C-E)** Representative immunofluorescence images of cells after phagocytosis for 1 h, fixation and staining with phalloidine-rhodamine (red) and DAPI (blue); C: No Fab, D: Fab C, E: Fab 452.

We have previously shown that CD13 can mediate phagocytosis in monocytic cells (30). To determine if CD13 is able to mediate phagocytosis also in human neutrophils, independently of other phagocytic receptors, we used a phagocytosis assay designed to promote the specific binding of the phagocytic prey to a defined receptor. With this assay, we analyzed phagocytosis mediated by FcγRII, FcγRIIIb or CD13 in human neutrophils using as phagocytic prey sheep erythrocytes labeled with CFSE and covered with F(ab’)_2_ fragments of goat anti-mouse IgG (EBS-Fab), as described in Materials and Methods. The results are shown in **Figures 2A, B**. The two distinct anti-CD13 Fabs that we used (Fab 452 and Fab C) were able to mediate phagocytosis of EBS-Fab through CD13 (percentage of CFSE-positive cells: Fab 452: 30.1% SEM ± 4.2; Fab C: 27.3%, SEM± 3.2). These percentages of CFSE positive cells are significantly different from the percentage of CFSE positive cells in the control (No Fab; mean 3.2%, SEM± 0.9). In samples incubated at 4°C, the percentages of CFSE-positive cells were virtually zero, regardless of incubating the cells with Fabs or no Fab (not shown). For comparison, we used the same selective phagocytosis assay to measure the phagocytosis through two receptors for IgG: FcγRII (mean 21.4%, SEM± 4.4) and FcγRIIIb (mean 23.3%, SEM± 4.0) (**Figure 2A**). The level of CD13-mediated phagocytosis is very similar to the phagocytosis mediated by FcγRII and FcγRIIIb, and no significant difference was found among phagocytosis mediated by FcγRs and CD13. To corroborate that CFSE positivity was due to internalized EBS-Fab, after the phagocytosis assay we took aliquots of the cells and stained the nucleus (with DAPI) and the cytoskeleton (with Phalloidin to visualize actin filaments). As shown in **Figures 2D, E**, a percentage of cells preincubated with Fab C or Fab 452 and incubated with EBS-Fabs showed CFSE fluorescence localized in specific and clearly defined regions in the cytoplasm. In contrast, intracellular CFSE fluorescence was not observed in the condition of No Fab (**Figure 2C**). These results demonstrate that in human neutrophils, CD13 is able to mediate phagocytosis to levels similar as those observed for phagocytosis through well-characterized phagocytic receptors such as FcγRII and FcγRIIIb.

### CD13-mediated phagocytosis requires actin polymerization and is dependent on Syk and PI3-K activity

**Figure 3:**
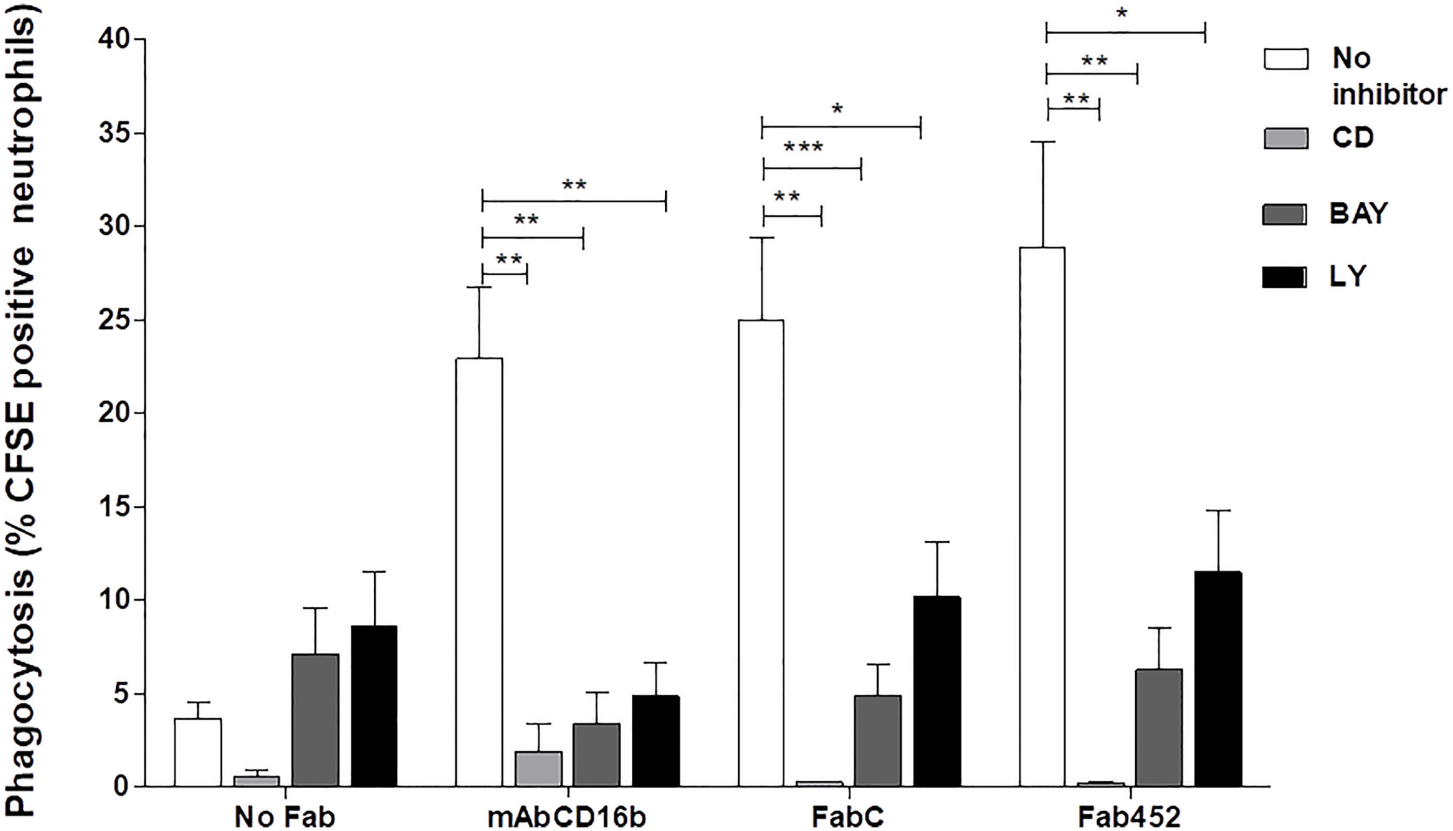
CD13-mediated phagocytosis requires actin reorganization and Syk and PIK3 activities. Neutrophils were incubated with 10 µg Fab fragments of mAb452 or mAb C (anti-CD13), with 10 µg of mAb G8 (anti-FcγRIIIb), or without antibody (no Fab) for 30 minutes at 4°C. After washing, cells were incubated with 5 µM cytochalasin D, 10 µM BAY, or 20 µM LY294002, or DMSO as vehicle control for 20 min at 37°C. Then, cells were incubated for 1 hour at 37°C with EBS-Fab. Non ingested erythrocytes were lysed and samples were analyzed by flow cytometry to determine the percentages of CFSE-positive cells. The bars show the mean ± SEM of CFSE-positive cells from 5 independent experiments for cytochalasin D, and from 11 experiments for the pharmacological inhibitors of kinases (BAY and LY294002). *p<0.05, **p<0.01, ***p<0.001.

Phagocytosis is characterized by the requirement of actin cytoskeleton rearrangement for formation of the phagocytic cup (37). To determine whether CD13-mediated particle internalization is dependent on actin cytoskeleton rearrangements, we evaluated CD13-mediated phagocytosis in neutrophils treated with 5 µM cytochalasin D, an inhibitor of actin polymerization. Treatment of cells with cytochalasin D resulted in a complete ablation of CD13-mediated phagocytosis (mean % of CFSE positive cells= 0.20%), compared with the phagocytosis observed in absence of cytochalasin D (mean = 25.0% with Fab C and 29% with Fab 452) (**Figure 3**). As expected, FcγRIIIb-mediated phagocytosis was also abrogated after cytochalasin D treatment of the cells (mean 2.32%). These data demonstrate that CD13-mediated phagocytosis depends on reorganization of the actin cytoskeleton.

Spleen tyrosine kinase (Syk) is known to play a crucial role in signaling by various immune receptors, such as the BCR, receptors for the Fc portion of antibodies, C-type lectins, etc. (38, 39). In neutrophils and macrophages, Syk is essential for activation of downstream signalling molecules (e.g., phospholipase Cγ1 and phosphatidylinositol 3-kinase (PI3-K)) during phagocytosis mediated by FcγR and other receptors (37, 39). Therefore, we evaluated whether Sky participates in the signaling cascade involved in CD13-mediated phagocytosis in human neutrophils using a highly selective Syk inhibitor, BAY613606. After incubating the cells with the corresponding Fab fragments and washing, we incubated neutrophils in the presence of 10 µM BAY for 30 minutes, and carried out the selective phagocytosis assay as described above. Treatment of cells with BAY resulted in a significant inhibition of CD13-mediated, as well as FcγRIIIb-mediated phagocytosis (**Figure 3**). This inhibition is evident for phagocytosis mediated by either of the two anti-CD13 Fabs used (C or 452). Treatment with BAY decreases the phagocytosis mediated by CD13 or by FcγRIIIb to levels similar to the non-specific phagocytosis (No Fab). These results suggest that in human neutrophils, CD13-mediated phagocytosis is dependent on Syk activity. To corroborate Syk activation induced by CD13 mediated phagocytosis, we evaluated Syk phosphorylation by intracellular staining and flow cytometry. Neutrophils were stimulated as for selective phagocytosis, and at 20 seconds, 1 and 2 minutes after stimulation at 37° C, cells were fixed, permeabilized and stained using a PE-labeled antibody that recognizes the phosphorylated Tyr 348 on Syk. The results show that, compared to control cells, there is an increase in phosphorylated Syk after the phagocytic stimulus (**Supplementary Figure 1**).

Phosphatidylinositol 3 kinase is a target molecule of Syk and its activation is essential for phagocytosis through different receptors. Also, PI3-K is involved in CD13-mediated homotypic cell adhesion of monocytic cells (15). Thus, we evaluated the participation of PI3-K in CD13-mediated phagocytosis in neutrophils. For this, we incubated cells with the PI3-K inhibitor LY294002 (20 µM) prior to the phagocytosis assay. CD13-mediated phagocytosis in cells treated with LY294002 was significantly inhibited as compared to phagocytosis in untreated cells. As expected, phagocytosis through FcγRIIIb was also inhibited in the presence of LY294002. These results show that PI3-K plays an important role in CD13-mediated phagocytosis (**Figure 3**).

### CD13-mediated phagocytosis is associated with production of reactive oxygen species in human neutrophils

**Figure 4:**
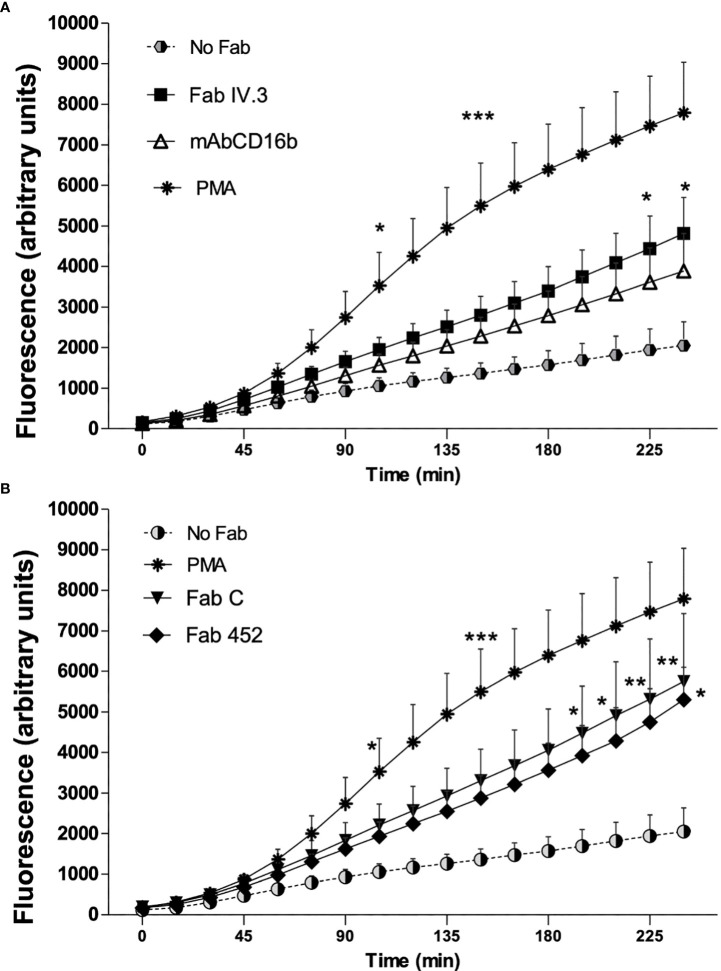
Production of reactive oxygen species associated to phagocytosis mediated by CD13 and FcγR in neutrophils. Human neutrophils were preincubated on ice with Fab fragments of anti-FcγRII (Fab VI.3) or Fab fragments of anti-CD13 (Fab 452 or Fab C) or mAb anti FcγRIIIb (CD16b) or no treatment (No Fab). The cells were washed and loaded with carboxy-H2DFFDA, for 30 min at 37°C. After washing, the cells were mixed with EBS-Fab not labeled with CFSE at a ratio of 20 EBS-Fab to 1 cell, in wells of black 96-well Inmuno Plates, and the fluorescence from oxidized carboxy-H2DFFDA was determined immediately and every 15 min as described in Materials and Methods. Results are expressed as mean ± SEM of 7 independent experiments, except for Fab 452 where n=5. Stimulation with PMA was used as a positive control. Statistical significance was computed through a one-way ANOVA with a Tukey *post hoc* test. *p<0.05, **p<0.01, ***p<0.001. **A** and **B** graphs display data from the same experiments, but are shown separately for clarity.

Production of reactive oxygen species (ROS) is an important microbicidal mechanism of neutrophils. To determine if CD13-mediated phagocytosis is associated with ROS production, cells were incubated on ice with Fab fragments of anti-FcγRII (Fab IV.3), or fragments of anti-CD13 (Fab 452 or Fab C), or mAb anti-FcγRIIIb (mAb CD16b), or no treatment (No Fab). After this, cells were washed and loaded with carboxy-H2DFFDA (a ROS-sensitive fluorescent dye). After washing, cells were incubated with EBS-Fab not labeled with CFSE, so as to replicate the stimulation used to evaluate phagocytosis in **Figure 2**. Every condition was assayed in triplicate wells. As a positive control for ROS production, neutrophils were stimulated with PMA. Fluorescence intensity from oxidized carboxy-H2DFFDA was determined immediately (initial reading) and every 15 min thereafter, during 240 min of incubation at 37°C. **Figure 4** shows the mean ± SEM of ROS production associated with phagocytosis by human neutrophils from seven individual donors. As shown, all stimuli induced a significant increase in ROS production, albeit with different time course. Stimulation with PMA induced production of ROS that became significantly different from unstimulated cells after 105 minutes. As expected, phagocytosis through FcγRII or FcγRIIIb induces a time-dependent increase in ROS production (**Figure 4A**). Phagocytosis mediated through CD13 is also associated with an increase in ROS production, irrespective of the mAb used (mAb C or mAb 452) to target the EBS-Fab to CD13 (**Figure 4B**), and the levels of ROS produced during phagocytosis through CD13 are similar to those induced during phagocytosis of FcγRs. These results suggest that phagocytosis through CD13 in human neutrophils generate ROS which could potentially promote the degradation of ingested material.

### CD13 crosslinking induces formation of neutrophil extracellular traps and enhances NETs release induced by PMA and IL-8

**Figure 5:**
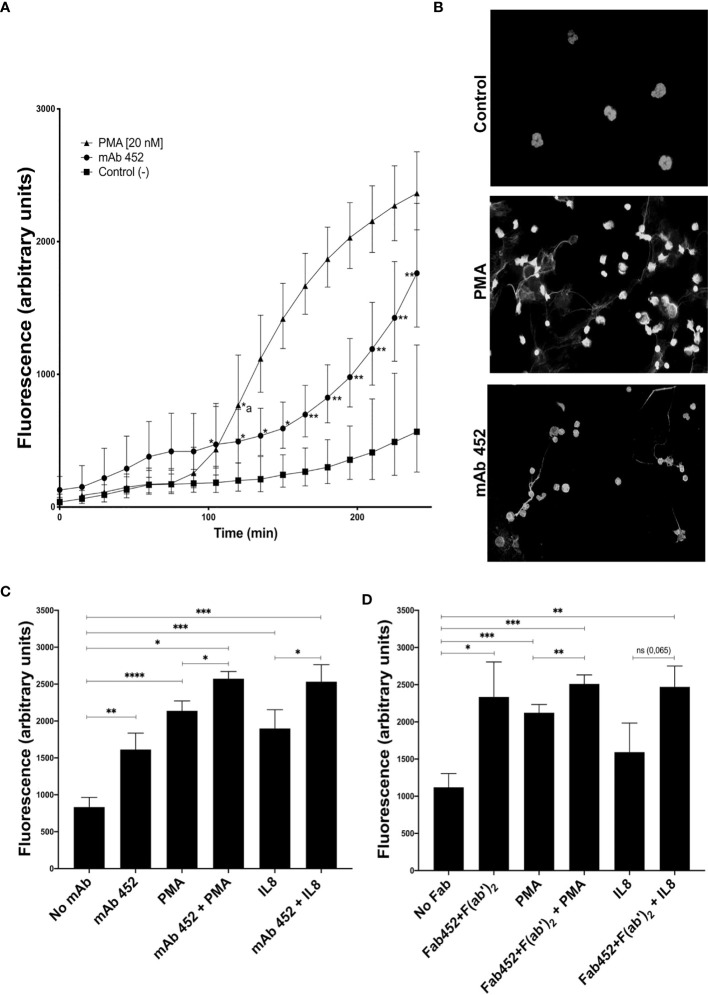
CD13 crosslinking induces neutrophil extracellular traps (NETs). Neutrophils were left untreated (control) or were incubated with mAb 452 or its Fab fragments for 30 minutes, washed, and stimulated with PMA (20 nM) or IL-8 (10 ng/ml) for 240 minutes, and fluorescence of extracellular DNA was evaluated with SYTOX-Green. **(A)** Time-course of SYTOX-Green fluorescence in cells stimulated with PMA, mAb 452 (anti-CD13) or non-stimulated. Fluorescence values are the mean ± SEM of 6 independent experiments. Statistical significance was calculated using one-way ANOVA with Tukey’s *post hoc* test (*p< 0.05, **p< 0.01) (*a: in the PMA curve, values are significantly different from control from 120 minutes onwards). **(B)** Immunofluorescence images of neutrophils stimulated with PMA, mAb 452 or non-stimulated for 240 min, fixed and incubated with Hoechst 33342^®^ to visualize extracellular DNA. **(C, D)** NETs release from human neutrophils stimulated for 240 min with mAb 452 **(C)**, or Fab 452 plus F(ab’)2 fragments of anti-mouse Ig **(D)**, PMA, or IL-8, or the combinations shown. Statistical significance was computed through a one-way ANOVA with a Tukey *post hoc* test. *p<0.05, **p<0.01, ***p<0.001, ****p<0.001.

To determine the ability of CD13 to stimulate another effector function of neutrophils, we evaluated if CD13 crosslinking induces NETs release using the cell-impermeable dye SYTOX-Green^®^. Cells were stimulated with mAb anti-CD13, with PMA as positive control, of left unstimulated. The time-dependent increase in fluorescence of SYTOX-Green^®^ was followed. **Figure 5A** shows the release of NETs by stimulated with PMA or with mAb 452. PMA induced an increase in SYTOX-Green fluorescence which becomes significant after 120 minutes, and continues increasing up to 240 minutes. Stimulation through CD13 by complete mAb452, induces an increase in SYTOX-Green fluorescence that is evident after 105 minutes (p<0.05), and becomes more significantly different (p<0.01) from control cells at 165 min. In order to evaluate if the observed increases in SYTOX-Green^®^ fluorescence are indicative of NET release, we stimulated cells on coverslips with the same stimuli and, after fixation, stained the DNA with Hoechst 33342^®^. As shown in **Figure 5B**, samples stimulated by either PMA or mAb 452 show the release of DNA filaments characteristic of NETs, in contrast to non-stimulated cells.

When cells were stimulated simultaneously with PMA and mAb452, the increase in fluorescence is higher and significantly different from the increases induced by each stimulus separately. As expected, stimulation of neutrophils with IL-8 (10 ng/mL), also induced an increase in SYTOX-Green fluorescence, and simultaneous crosslinking of CD13 resulted in a significantly higher fluorescence as compared to IL-8 stimulation alone (**Figure 5C**). Cell stimulation with Fab fragments of mAb 452 plus secondary F(ab’)_2_ fragments of anti-mouse Ig yielded similar results (**Figure 5D**), in that the crosslinking of CD13 induces a significant increase in fluorescence compared to unstimulated cells, and that stimulation through CD13 and PMA simultaneously, significantly increases the response induced by each stimulus separately. We also observed an enhancing effect of CD13 crosslinking in the fluorescence observed after stimulation by IL-8, but the increase was not statistically significant (**Figure 5D**).

**Figure 6:**
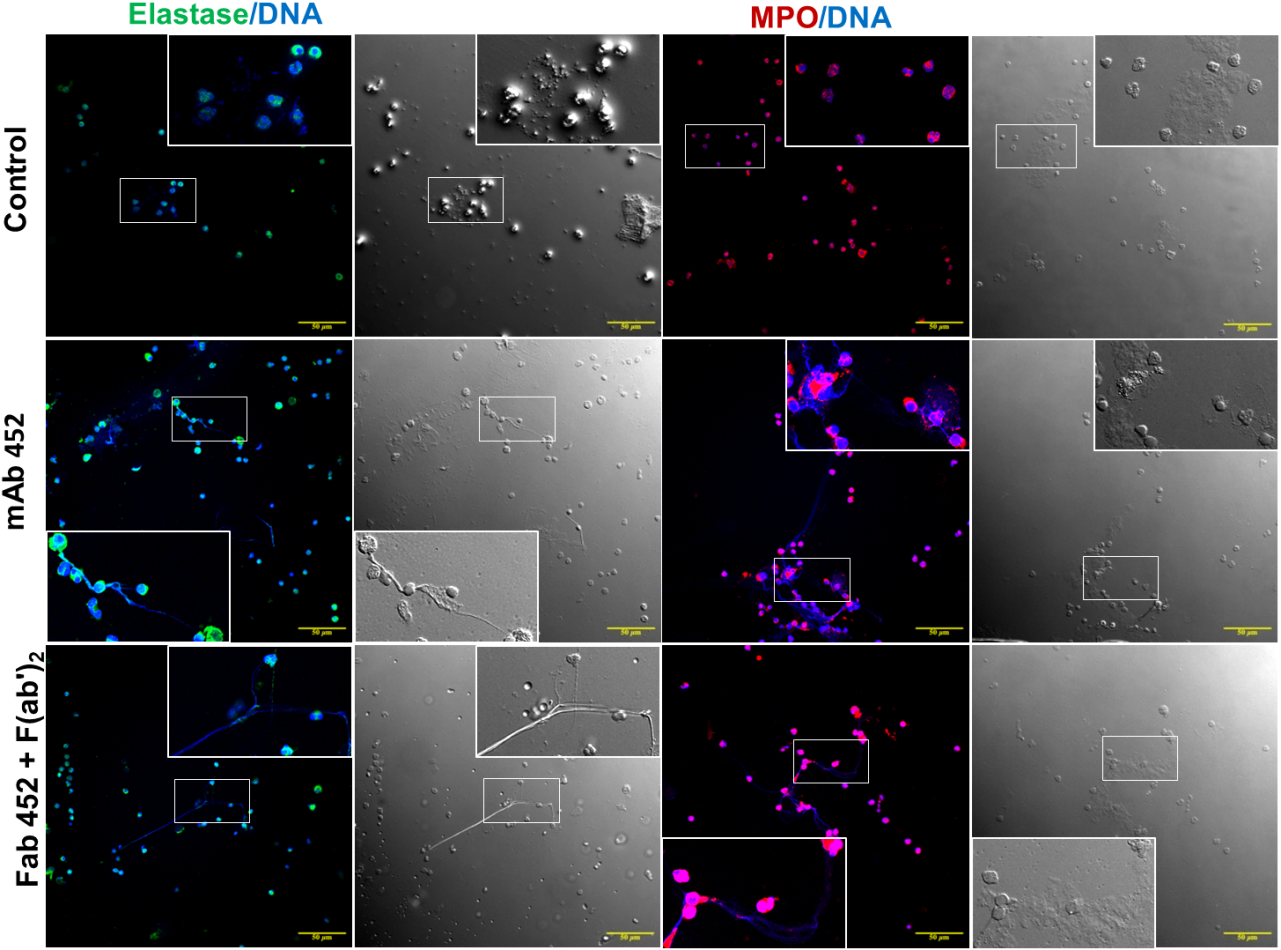
Myeloperoxidase and Neutrophil elastase are associated with extracellular DNA released after CD13 crosslinking. Confocal microscopy images of human neutrophils stimulated for 240 min with mAb 452, or Fab 452 plus F(ab’)2 fragments of anti-mouse Ig, or left unstimulated (control). Cells were fixed and stained to visualize DNA (blue), myeloperoxidase (red) or neutrophil elastase (green). Each immunofluorescence image is shown together with its respective bright field micrograph. Inserts show an enlargement of a selected area of each image. Bars: 50 µm.

To further support that the extracellular DNA is indeed forming NETs, we stimulated cells and after fixation, stained the preparation for neutrophil elastase (NE) and myeloperoxidase (MPO), two proteins considered as canonical markers of neutrophil NETs (40), and stained DNA with Hoechst 33342^®^. Representative confocal images are shown in **Figure 6**. Unstimulated cells have a well-defined morphology, and both MPO and NE are located in the cytoplasm. In contrast, after cell stimulation through CD13 both MPO and NE could be found associated to the extracellular DNA. These results strongly suggest that neutrophil stimulation through CD13 induces the release of NETs.

### CD13 crosslinking induces secretion of pro-inflammatory cytokines in human neutrophils

**Figure 7:**
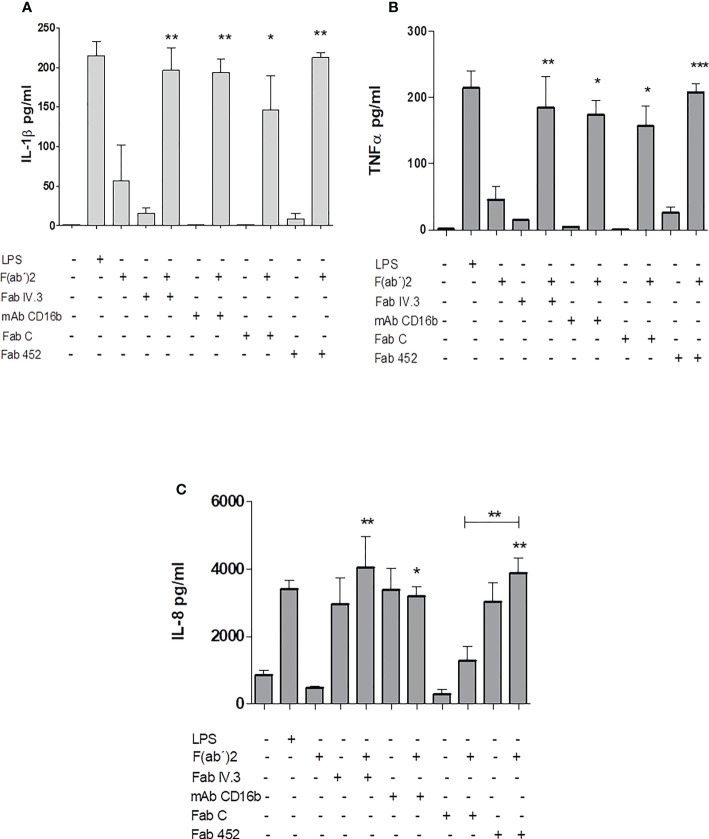
Production of inflammatory cytokines by Neutrophils stimulated by CD13 crosslinking. Neutrophils were incubated with Fab fragments of anti-CD13 mAbs (452 or C), Fab fragments of anti-FcγRII (Fab IV.3), or with anti- FcγRIIIb mAb (mAb CD16b) with or without secondary F(ab’)2 fragments of goat anti-mouse Ig, as indicated. As control, cells were stimulated with LPS (positive control) or with F(ab’)2 fragments alone. The cells were incubated for 24 hours and the cell culture supernatants were recovered. Concentrations of IL-1β **(A)**, TNF-α **(B)** and IL-8 **(C)** in the supernatants were quantitated by a multiplex array. Results are expressed as mean ± SEM of 3 independent experiments using cells from 3 different donors. Statistical significance was calculated using one-way ANOVA with Tukey *post hoc* test (**p< 0.01, ***p< 0.001). Asterisks shown above each bar indicate statistical difference as compared to cytokine production by cells incubated with F(ab’)2 fragments of anti-mouse Ig alone (third bar in each graph). In **(C)**, asterisks (**) above the line (
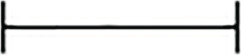
) drawn above the bar for Fab452 alone (penultimate bar) refer to the comparison between FabC plus secondary F(ab’)2 fragments and the Fab452 plus secondary F(ab’)2 fragments, that show a statistically significant difference between them. *p<0.05.

To analyze the secretion of pro-inflammatory cytokines by human neutrophils stimulated by CD13 crosslinking, cells were incubated for 30 minutes with Fab fragments of anti-CD13 mAbs (FabC or Fab452), or anti-FcγRII (FabIV.3), or mAb anti-CD16b (mAb 3G8), or left untreated. After washing, cells were treated with secondary F(ab’)2 fragments of anti-mouse-Ig, and samples of cells not incubated with Fabs were treated with LPS (from *E. coli*, 100 ng/mL) as a control. Cells were incubated for 24 h at 37°C. Cell-free culture supernatants were harvested and stored at -20°C, until they were used for determination of the production of IL-1β, TNF-α, IL-8, IL-6 and IL-10. The results are shown in **Figure 7**. Crosslinking of CD13, as well as of FcγRII and FcγRIIIb induces the secretion of IL-1β, TNF-α, and IL-8, and the levels of cytokine secreted are similar as those induced by stimulation with LPS. Secretion of IL-1β and TNF-α show similar requirements for secretion, in that cytokine secretion is only observed when the primary specific antibodies are crosslinked by a secondary anti mouse Ig. Interestingly, IL-8 secretion showed a different pattern: on the one hand, of the two anti-CD13 Fabs employed, only Fab 452 (but not Fab C) induced IL-8 secretion. Also, it is interesting that Fab 452, Fab IV.3 and mAb CD16b, induced IL-8 secretion in the absence of secondary F(ab’)2 fragments of anti-mouse-Ig. Although some secretion of IL-6 was also observed after stimulation through CD13 and FcγRs, the results were not statistically different from the control (not shown). Also, no differences were observed in IL-10 secretion among the different experimental conditions examined (not shown). These results indicate that the crosslinking of CD13 in human neutrophils induce the release of the proinflammatory cytokines IL-1β, TNF-α, and IL-8.

## Discussion

Neutrophils are the most abundant circulating leukocytes in humans and they participate in the first line of defense against invading pathogens (2). Neutrophils can be activated through direct recognition of Pathogen-associated molecular patterns (PAMPs) or Damage-associated molecular patterns (DAMPs), by different pattern-recognition receptors such as TLRs, C-type lectins, or scavenger receptors, among others; or by interaction with opsonized particles or microorganisms through opsonic receptors such as receptors for complement fragments and receptors for the Fc portion of immunoglobulin G (FcγRs). This recognition triggers several effector mechanisms deployed by these cells (7, 41). However, neutrophils express on its membrane other proteins whose roles in triggering or modulating effector functions of these cells are unknown.

CD13 was originally identified as a surface protein in myeloid cells (42). Later, it was realized that CD13 is the enzyme Aminopeptidase N, whose role in regulation of the activity of bioactive peptides like enkephalins, bradikinin, angiotensin, etc. had been characterized. CD13/APN is expressed in several cells and tissues such as kidney, intestine, liver and placenta. CD13 has also been shown to be expressed in vascular endothelium and to participate in angiogenesis (43). Besides its function as a peptidase, CD13 was shown to participate in adhesion processes of monocytic cells, independently of its enzymatic activity (15, 16), and thus, efforts were made to elucidate the possible participation of CD13 in other functions of monocytes/macrophages. So far, a role for CD13 has been shown in several different functions such as adhesion, phagocytosis and ROS production in monocytes/macrophages (16, 30, 36, 44). However, its ability to participate in the different effector functions of neutrophils has not been reported.

Human neutrophils are known to express CD13 (31, 45). As expected, more than 98% of neutrophils from all human donors that we analyzed (>30) express CD13 on their membrane, although some variation was found in the level of CD13 expression between donors (**Figure 1A**). In the same samples, we measured the expression of CD16b, a low affinity receptor for IgG that is expressed exclusively in neutrophils. Expression of CD16b in >98% of cells indicates that the populations of cells that we used for our experiments were not contaminated with other leukocytes. Also, since practically all CD16b+ cells are also CD13+, this corroborates that CD13 is expressed in all human neutrophils, as has been reported. Although all Neutrophils express CD13, the expression level was variable between donors (**Figure 1B**). This might reflect variations in the physiological status of individuals at the moment of blood withdrawal, as the level of CD13 expression in human Neutrophils has been shown to be modulated by certain stimuli, such as IL-8, fMLP, and others (31, 32).

Based on our previous studies of CD13 involvement in effector functions of myelomonocytic cells, we hypothesized that CD13 may be involved in different neutrophil effector functions. Phagocytosis is the hallmark of specialized cells, including neutrophils, to eliminate microorganisms. Neutrophils can internalize both opsonized and non-opsonized particles. The principal opsonin receptors of neutrophils are Fcγ receptors and a subgroup of β2 integrins, which bind to immunoglobulin G or complement-coated particles, respectively (1, 46–48). In order to evaluate if CD13 can act as a primary phagocytic receptor in Neutrophils, we used an experimental system through which CFSE-labeled sheep erythrocytes (as phagocytic preys) are targeted to specific proteins expressed on the membrane of the phagocyte. This is accomplished by allowing the binding of murine monoclonal antibodies (or their Fab fragments) specific for the membrane protein, to the cells, and incubating the cells with bound antibody with erythrocytes that carry on their membrane F(ab’)2 fragments of anti-mouse Ig antibodies. In this way, the phagocytic prey interacts with the phagocyte specifically through the membrane protein of interest (30, 36). The main phagocytic Fc receptors of human resting neutrophils are FcγRIIa (CD32) and FcγRIIIb (CD16b) (37, 49) and phagocytosis through these receptors has been well characterized. Thus, we evaluated in parallel phagocytosis through CD13 (using two different Fabs) and through both FcγRIIA (using Fab fragments of mAb IV.3) and FcγRIIIb (using mAb 3G8). The results showed that CD13 is able to mediate phagocytosis, and the level of phagocytosis observed is similar to that mediated by either FcγRII or FcγRIIIb. Phagocytosis is characterized by actin-dependent extension of the plasma membrane around the particle (phagocytic cup) (50, 51). We demonstrated that CD13-mediated phagocytosis in neutrophils is completely abolished by treatment with cytochalasin D, an inhibitor of actin filaments formation and elongation. Moreover, parallel samples maintained at 4°C, showed no cell-associated fluorescence. Together, these results indicate that the observed CD13-mediated internalization of EBS is indeed a phagocytic process. A similar dependence on actin rearrangement and temperature was previously shown for CD13-mediated phagocytosis in THP-1 monocytic cells (30).

Phagocytosis through FcγRs requires phosphorylation of tandem tyrosine residues within the context of an immunoreceptor tyrosine activation motif or ITAM. Phosphorylation of the ITAM motifs in turn serves to generate docking sites for proteins bearing SH2 domains, particularly the tyrosine kinase Syk (52). Syk activity is essential for Fc-mediated phagocytosis, as neutrophils from Syk-deficient mice are unable to ingest IgG opsonized particles (53). Other phagocytic receptors such as CD36, MARCO and β1 integrins also require Syk activity for phagocytosis (54, 55). Syk activation is accompanied by stimulation of phosphatidylinositol 3-kinase (PI3-K), which is largely responsible for converting phosphatidylinositol 4,5-bisphosphate (PI4,5P_2_) to phosphatidylinositol 3,4,5-trisphosphate (PI3,4,5P_3_). Activation of PI3-K is essential for initiating cytoskeleton rearrangements necessary for formation of the phagocytic cup (50, 52, 53, 56, 57). Syk-deficient mice show reduced PI3-K phosphorylation, implying that activation of the tyrosine kinase is an early event that contributes indirectly to phosphoinositide metabolism. Our results indicate that CD13-mediated phagocytosis is dependent on Syk and PI3-K enzymatic activities, as pretreatment of human neutrophils with BAY or with LY294002 resulted in an abatement of phagocytosis through CD13. These findings are similar to those observed in monocytes (30), and suggest that in neutrophils, Syk and PI3-K are activated by crosslinking CD13, and their activities are important for phagocytosis through this receptor. The signaling pathway triggered after activation of CD13 is at present not fully described; however, this study demonstrates the importance of Syk and PI3-K in the phagocytosis mediated by CD13.

Closure of the phagocytic cup is followed by assembly of the NADPH-oxidase complex on the phagosome membrane, which initiate the production of superoxide and other microbicidal species. We therefore evaluated the production of ROS coupled to phagocytosis mediated by CD13. Production of ROS is an essential function for efficient killing of pathogens after phagocytosis (58). We found that phagocytosis mediated by CD13 is coupled to ROS production in human neutrophils, and the amount of ROS produced is similar to that induced by phagocytosis through FcγRIIa and FcγRIIIb. PMA stimulation of Neutrophils induced ROS production with a time-course that is different from that observed associated with phagocytosis. The major source of ROS in neutrophils is the phagocyte NADPH oxidase (NOX2). This enzymatic complex requires a “priming” signal, e.g. activation of a receptor (59, 60). Priming facilitates assembly of NADPH oxidase *via* mobilization and phosphorylation of granular and cytosolic components to the phagosomal membrane (60, 61). Thus, the differences in the time course of ROS production induced by PMA or phagocytosis could be related to the fact that PMA directly activates protein kinase C, which activates p47^phox^ triggering the assembly of NADPH oxidase complex (62), while phagocytosis requires a series of signal transduction events involving Syk, PLC-γ, Ca^+2^ mobilization, cytoskeleton rearrangements, that need to occur prior to initiation of the assembly of the oxidase complex on the phagosome membrane (63).

No significant differences were found between the two different anti-CD13 Fabs (C and 452), for phagocytosis and ROS production. However, mAb 452-mediated phagocytosis was usually higher than that mediated by Fab C, while ROS production mediated by Fab C was usually higher than that mediated by Fab 452. This could be related to the different epitopes recognized by each mAb. Previously, we have reported that mAb C and mAb 452 had different abilities to promote homotypic aggregation of monocytes (33).

The phagocyte NADPH oxidase (NOX2) produces ${\text{O}}_{2}^{-}$ and its secondary metabolites (H_2_O_2_, OH^-^), that are considered both as effector molecules for killing of microorganisms, but also as second messengers that can induce NET formation, necroptosis or apoptosis (64). Several stimuli, including invading microorganisms such as bacteria, fungi, viruses and protozoa, but also internal stimulii such as IL-8, H_2_O_2_, platelets, autoantibodies or complement fragments, induce the release of NETs (65). These are large three-dimensional structures consisting of chromatin fibers with several proteins attached, such as histones, neutrophil elastase, myeloperoxidase, cathepsins, and defensins, among others (66). Although initially known for their antimicrobial activities, NETs are known to participate in other functions like inflammation and thrombosis (67). Different receptors such as TLRs, integrins, and FcγRs are capable of inducing NETs (66, 68) through different mechanisms (69–72), and signaling pathways (72). Our results show that crosslinking of CD13 with specific monoclonal antibodies on human neutrophils induces the release of NETs. Similar to what we observed for ROS production, the time-courses of NET release induced by PMA or by crosslinking of CD13 were different. While NETs release by PMA was significant after 105 minutes and from there it shows a steep increase, the release observed after CD13 crosslinking showed a slower rise, and after incubation for 4h it did not reach the levels observed 4h after PMA stimulation. Unfortunately, after 4 hours the viability of the neutrophils starts to decline sharply and longer experiments are unreliable. However, the demonstration that NETs induced by CD13 crosslinking contain both neutrophil elastase and myeloperoxidase supports the conclusion that the DNA structures observed by microscopy are indeed NETs. As has been previously reported, stimulation of neutrophils with IL-8 induces release of NETs (66, 73). The CXCR1/2 (IL-8 receptor) is a G-protein coupled receptor that activate PI3-K and Ras protein pathways to induce release of NETs (74). It is interesting that crosslinking of CD13 simultaneously with IL-8 or PMA stimulation results in an increased release of NETs. This suggests that *in vivo*, cooperative stimulation by distinct receptors could result in enhanced microbial killing.

Release of NETs and phagocytosis have been seen as antagonistic or at least mutually excluding responses, with one event negatively regulating the other, leading to the proposal that neutrophils must integrate several signals to make a choice between phagocytosis vs NET formation (75). The observation that CD13 is able to mediate both phagocytosis and NETs formation does not imply that they can do so simultaneously or sequentially. Since we stimulated NETs release by crosslinking CD13 with soluble antibodies and not by a phagocytic stimulus, we could not determine if neutrophils could release NETs after having phagocytized through CD13. As reviewed by Chen et al. (65), it is known that a series of well-known phagocytic receptors have also been shown to trigger NETs release, such as the complement receptor CR3 (Mac-1 or CD11b/CD18) and Fc receptors. Our results suggest that CD13 could be added to this list.

The results presented in this paper, extend our previous reports of CD13 mediating phagocytosis, ROS production and cytokine secretion in human monocytes and macrophages (30, 36, 76), and support the hypothesis that CD13 can be considered an immune receptor of myeloid cells. Structurally, CD13 has a large extracellular region (>900 aa residues), which could potentially contain distinct sites involved in different functions. Besides the enzymatic active site which has been precisely defined, CD13 provides a large exposed surface that could be involved in binding different ligands that could stimulate one or more of the many functions in which this molecule has been shown to participate (25). While some of these functions could require CD13 crosslinking to initiate signal transduction, others could require binding to a specific region of CD13. In fact, it has been shown that different CD13-specific mAbs that bind to distinct epitopes on CD13 have opposite consequences for cell adhesion (33). Moreover, membrane CD13 is heavily glycosylated, and specific carbohydrate moieties could be involved in binding of some ligands, as has been shown for CD13 interaction with E-selectin (77). Until now, monoclonal antibodies specific for CD13 have been used as surrogate ligands to induce CD13 crosslinking and bring about signal transduction and effector functions. Undoubtedly, identification of natural ligands that can trigger CD13-mediated effector functions *in vivo* is of utmost importance to definitively establish CD13 as an immune receptor. Despite efforts in several laboratories, it is still unclear which are the CD13 ligand(s) that are able to induce the responses induced by crosslinking with specific antibodies. Among the proposed candidates, Galectin-3 (Gal-3), a β-galactoside binding lectin, is an interesting possibility. Direct interaction of Gal-3 with CD13 has been shown by Yang et al. (78) using a human cDNA phage display biopanning method to identify proteins interacting with CD13. Also, Gal-3 interaction with CD13 was found in a study that used affinity chromatography for identification of Gal-3 ligands in seminal fluid (79). Furthermore, Gal-3 was also shown to coimmunoprecipitate with CD13 (80). On the other hand, extracellular Gal-3 has been shown to participate in several immune-related functions, including immunity against pathogens as well as acute and chronic inflammation. Moreover, Gal-3 has been shown to bind to the surface of several bacteria, fungi and parasites, and to have pro-inflammatory properties promoting the infiltration of neutrophils and other immune cells to infected sites (reviewed in (81, 82). However, ascribing the reported effects of Gal-3 in infections to its binding to CD13 on myeloid cells is difficult, because potential saccharide ligands for Gal-3 are present on various different glycoproteins on the membrane of these cells.

Also, a more complete delineation of the signal transduction pathways involved in cellular functions initiated through CD13 is necessary. Several proteins that participate in effector functions triggered by activating FcγRs, such as Src kinases, Syk, and PI3-K, have been shown to participate in signal transduction initiated by CD13. It is interesting that it was shown that phagocytosis and ROS production mediated by FcγRs and by CD13 are affected in similar ways by the polarization state of human macrophages (36), suggesting at least some degree of parallelism between the pathways initiated by CD13 and FcγRs.

In summary, we have shown that CD13 is able to activate key effector functions of human neutrophils, at levels that are similar to those attained after activation of the same functions by FcγRs. Activation of these functions are dependent on activation of Syk and PI3-K. Pending identification of natural CD13 ligands, these results support that a role as an innate immune receptor could be added to the functions in which the moonlighting protein CD13 has been shown to participate.

## Data availability statement

The raw data supporting the conclusions of this article will be made available by the authors, without undue reservation.

## Author contributions

EP-F and EO conceived and designed the experiments, analyzed the results, and wrote the manuscript. PA-C designed and performed the NETs experiments. EP-F performed the experiments and wrote the first draft of the manuscript. All authors contributed to revision of the final version of the manuscript.

## Funding

This work was supported by research grants from Dirección General de Asuntos del Personal Académico (DGAPA)-UNAM (PAPIIT- IN205617 and IN208320) and CONACYT (252428). EP-F was supported by a postdoctoral fellowship from the Programa de Becas Posdoctorales de Dirección General de Asuntos del Personal Académico (DGAPA) from the Universidad Nacional Autónoma de México.

## Acknowledgments

The authors thank all individuals who voluntarily agreed to donate blood samples for this study. They also thank Dr. Carlos Rosales (Instituto de Investigaciones Biomédicas, UNAM) for the donation of mAb anti-FcγRIIIb (3G8), and Dra. Marcela Valdés (Instituto Nacional de Psiquiatría, Mexico) and Dr. Miguel Tapia (Instituto de Investigaciones Bomédicas, UNAM) for help with immunofluorescence microscopy. PA-C thanks Dr Cesar Diaz Godinez for help with neutrophil isolation. We thank the Laboratorio Nacional de Citometría de Flujo (LabNalCit, Instituto de Investigaciones Biomédicas, UNAM-CONACYT) for assistance and advice with flow cytometry.

## Conflict of interest

The authors declare that the research was conducted in the absence of any commercial or financial relationships that could be construed as a potential conflict of interest.

## Publisher’s note

All claims expressed in this article are solely those of the authors and do not necessarily represent those of their affiliated organizations, or those of the publisher, the editors and the reviewers. Any product that may be evaluated in this article, or claim that may be made by its manufacturer, is not guaranteed or endorsed by the publisher.

## Abbreviations

CD13, Aminopeptidase N; CFSE, Carboxyfluorescein succinimidyl ester; DAG, Diacylglycerol; DAMPs, Damage-associated molecular patterns; EBS-Fab, CFSE-labeled sheep erythrocytes covered with F(ab)´2 goat anti-mouse Ig; FcγR, Receptor for the Fc portion of immunoglobulin G; fMLP, N-formyl-Met-Leu-Phe; HBSS, Hanks’ balances salt solution; MARCO, Macrophage receptor with collagenous structure; MFI, Mean fluorescence intensity; MPO, Myeloperoxidase; NE, Neutrophil Elastase; NETs, Neutrophil Extracellular Traps; NOX2, phagocyte NADPH oxidase; PAMPs, Pathogen-associated molecular patterns; PFA, Paraformaldehyde; PI3,4,5P3, Phosphatidylinositol 3,4,5-trisphosphate; PI3-K, Phosphatidylinositol 3-kinase; PI4,5P2, Phosphatidylinositol 4,5-bisphosphate; PLC, phospholipase C; PMA, Phorbol-12-Myristate-13-Acetate; PTEN, Phosphatase and tensin homolog; ROS, Reactive oxygen species; SRBCs, Sheep red blood cells; Syk, Spleen tyrosine kinase; TLRs, Toll-like receptors.
