# Supplementary material for: Crosslinking of membrane CD13 in human neutrophils mediates phagocytosis and production of reactive oxygen species, neutrophil extracellular traps and proinflammatory cytokines
Source: Front Immunol. 2022 Nov 10;13:994496. doi: 10.3389/fimmu.2022.994496 (PMC9686367; doi:10.3389/fimmu.2022.994496)
Supplement: Supplementary file 1 [file DataSheet_1.pdf]

Supplementary Figure 1

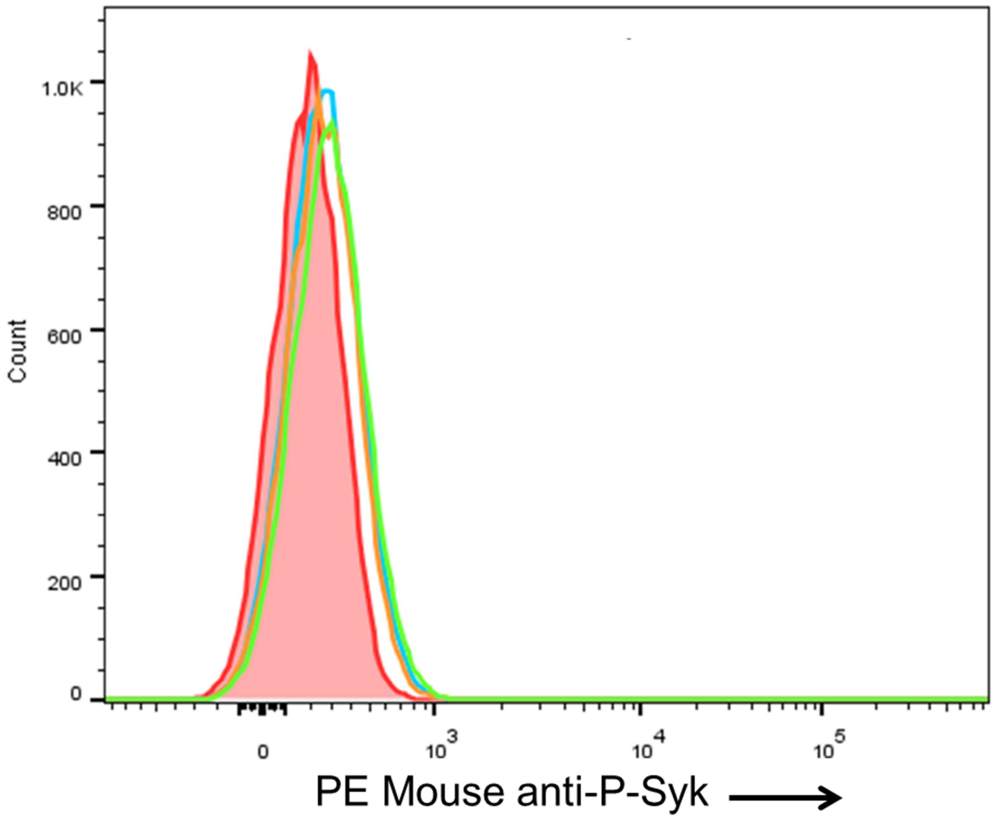

Supplementary Figure 1. Syk is phosphorylated during CD13-mediated phagocytosis. Erythrocytes (EBS-Fab) were prepared with biotin, streptavidin and biotinylated - goat anti mouse IgG F(ab')2 fragments, as described in material and methods, but without CFSE loading. Purified neutrophils were incubated for 30 min with Fab 452 (40 ug/ml) and washed. Aliquots with $0.25x10^6$ cells were mixed with EBS-Fab at a !:20 ratio. After adding the erythrocytes, samples were spun down and placed on ice for 20 min. After this time samples were incubated in a thermoblock at 37°C for 20 seconds (blue histogram), 1min (yellow histogram) or 2 min (green histogram), and treated with 4% paraformaldehyde to fix the cells. Control samples (red shaded histogram) were immediately fixed after incubation on ice. After fixation, cells were washed, erythrocytes were lysed by a hypotonic shock, and cells were permeabilized with BD Perm/Wash buffer for 40 minutes. After washing, cells were stained with PE-Mouse anti-phospho-Syk antibody (BD. Cat. 558529) for 30 min and read in the Attune® cytometer. Histograms from a representative experiment (n=4).
